# Interleukin-6 signaling pathway in Mendelian randomization: A 10-year bibliometric analysis

**DOI:** 10.1097/MD.0000000000037507

**Published:** 2024-04-05

**Authors:** Shaoze Jing, Jiani Wang, Shuhan Yang, Hua Wu

**Affiliations:** a Department of Orthopedics, Third Hospital of Shanxi Medical University, Shanxi Bethune Hospital, Shanxi Academy of Medical Sciences, Tongji Shanxi Hospital, Taiyuan, China; b Department of Pediatrics, Shanxi Medical University, Taiyuan, China.

**Keywords:** bibliometric analysis, interleukin-6, Mendelian randomization, visualization analysis, VOSviewer

## Abstract

Interleukin 6 (IL-6), a pleiotropic cytokine, is crucial in a variety of inflammatory and immunological disorders. In recent years, mendelian randomization, which is a widely used and successful method of analyzing causality, has recently been investigated for the relationship between the IL-6 pathway and related diseases. However, no studies have been conducted to review the research hotspots and trends in the field of IL-6 signaling pathway in Mendelian randomization. In this study, the Web of Science Core Collection (WoSCC) served as our literature source database to gather articles about the IL-6 signaling pathway in Mendelian randomization from 2013 to 2023. VOSviewer (version 1.6.18), Microsoft Excel 2021, and Scimago Graphica were employed for bibliometric and visualization analysis. A total of 164 documents that were written by 981 authors coming from 407 institutions across 41 countries and published in 107 journals were located from January 2013 to August 2023. With 64 and 25, respectively, England and the University of Bristol had the highest number of publications. Frontiers in Immunology is the most prolific journal, and Golam M Khandaker has published the highest number of significant articles. The most co-cited article was an article entitled the interleukin-6 receptor as a target for prevention of coronary-heart-disease: a Mendelian randomization analysis, written by Daniel I Swerdlow. The most popular keywords were “mendelian randomization,” “interleukin-6,” “il-6,” “c-reactive protein,” “association,” “coronary-heart-disease,” “inflammation,” “instruments,” “risk,” “rheumatoid arthritis,” “depression.” The full extent of the existing literature over the last 10 years is systematically revealed in this study, which can provide readers with a valuable reference for fully comprehending the research hotspots and trends in the field of IL-6 signaling pathway in Mendelian randomization.

## 1. Introduction

Interleukin 6 (IL-6) is a pleiotropic cytokine that undertakes multiple functions in the body. The first successful clone of the IL-6 gene was accomplished by Hirano T et al in 1986.^[1]^ After this achievement, fundamental research progressed quickly, and in the early 1990s, the entire image of the IL-6 signaling system was identified.^[2]^ The gene that codes for human IL-6 has been located on chromosome 7p21 and is made up of 184 amino acids, containing a signal peptide of 28 amino acids.^[3]^ Under inflammatory conditions, IL-6 synthesis and secretion are induced and are engaged in a variety of in vivo processes, including immunity and inflammation.^[4,5]^ IL-6 is highly associated with the progression of a variety of diseases, which association has been shown for the first time in cardiac mucinous tumors.^[6]^ Follow-up studies have also demonstrated that the dysregulation of IL-6 can occur in a variety of diseases including rheumatoid arthritis,^[7]^ systemic lupus erythematosus,^[8]^ inflammatory myopathies,^[9]^ systemic sclerosis,^[10]^ myeloma,^[11]^ and so on. In the diseases described above, IL-6 typically exerts its effects through 2 pathways, including the Janus kinase-STAT3 pathway and the Janus kinase-SHP-2-mitogen-activated protein kinase pathway.^[12,13]^

Mendelian randomization, a relatively recent method of causal inference, has recently assumed a more significant position in the field of observational epidemiology. Randomized controlled trials are known to be the gold standard for establishing causal analyses. However, they are not always feasible due to factors including cost, practicality, and even ethics.^[14]^ Mendelian randomization, a tool to identify causality by screening genetic variables, is now a time-dependent and cost-effective method. Mendelian randomization is a method of analysis in the field of econometrics which employs instrumental variables and the principle that genotypes are generally unaffected by confounding, reverse causal associations in populations, to indirectly determine whether observed associations between exposures and outcomes are consistent with causal associations. The aforementioned method of etiologic inference efficiently minimizes the influence of confounding factors from standard observational research, as well as the interference with reverse causation in the correlation effect.^[15]^

In recent years, mendelian randomization has been employed much more frequently lately to explore the IL-6 signaling pathway and related diseases. Multiple Mendelian randomization analyses have demonstrated the connection between IL-6 signaling pathway and various disorders, including Depression,^[16]^ rheumatoid arthritis,^[17]^ systemic lupus erythematosus,^[18]^ Breast Cancer,^[19]^ etc. However, to the best of our knowledge, there is currently no systematic analysis and summary in this area. In order to systematically examine the trends and hotspots of IL-6 signaling pathway in Mendelian randomization, in this study, we carried out a bibliometric analysis. By evaluating databases and literature characteristics, bibliometrics analysis is an invaluable instrument for evaluating trends in a field and identifying the most significant areas for future research.^[20]^ In the present study, we explored the current trends and hotspots of IL-6 signaling pathway in Mendelian randomization in the last 10 years from a bibliometric approach.

## 2. Materials and methods

Relevant publications were retrieved from the Web of Science Core Collection (WoSCC) adopting the search strategy provided in Table 1. The data was searched and exported as of August 23, 2023, to avoid conflicts brought on by database updating. The information retrieved is analyzed and presented graphically employing VOSviewer (version 1.6.18), Microsoft Excel 2021, and Scimago Graphica. VOSviewer was utilized to investigate countries, journals, keywords, years of publication, institutions, and authors of this field. Trends in publications over time were visualized with Microsoft Excel 2021. In addition, a world map of publications distributed in countries was drawn by Scimago Graphica.

**Table 1:** Summary of literature search strategy in this study.

Content	
Research database	Web of Science Core Collection
Citation indexes	SCI-EXPANDED
Searching period	January 2013 to August 2023
Language	“English”
Searching keywords	TS = (Interleukin-6 OR Interleukin 6 OR IL-6 OR IL 6) and TS = (Mendelian randomization)
Document types	“Articles” and “Review”
Date extraction	Export with full records and cited references in plain text format
Sample size	164

IL-6 = interleukin 6.

## 3. Results

### 3.1. General information

**Figure 1.**
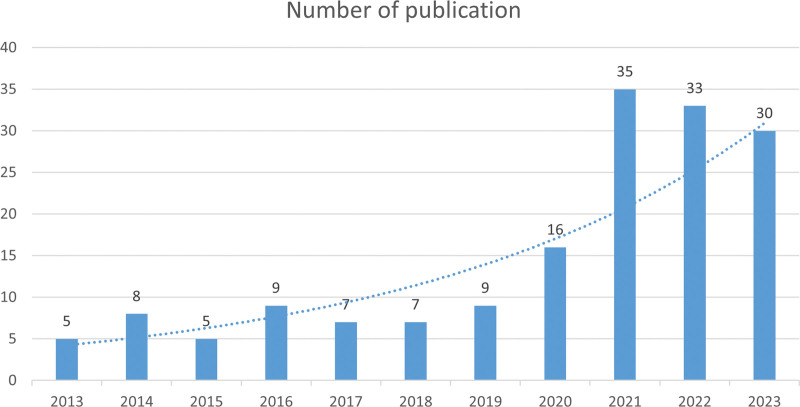
The annual trends of publications.

**Figure 2.**
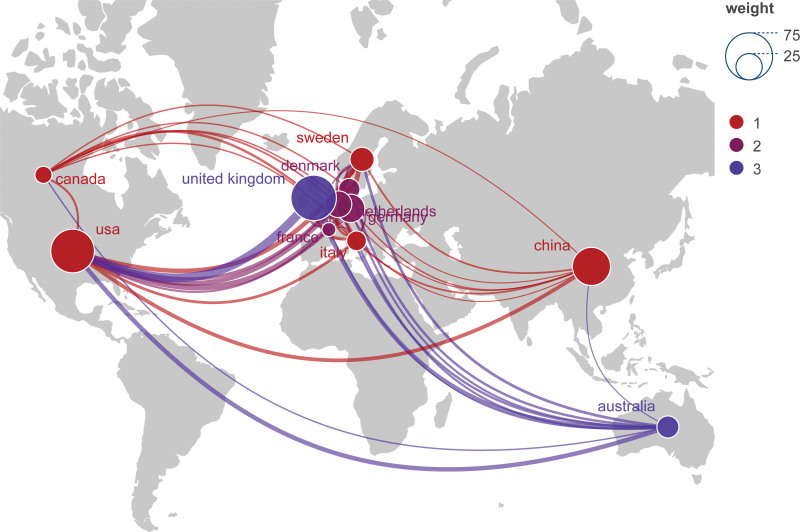
A world map of publications delivered in different countries (The size of the node is an indication of the number of publications).

According to the established search strategy, a total of 164 documents that were written by 981 authors coming from 407 institutions across 41 countries and published in 107 journals were located from January 2013 to August 2023. The details on trends in publications over time are displayed in Figure 1. From 2013 to 2019, publications in the field of IL-6 signaling pathway in Mendelian randomization offer a steady development, whereas the quantity of documents related to this field has significantly increased since 2020. Furthermore, Figure 2 displays a world map of publications delivered in different countries. In terms of publications in this field of IL-6 signaling pathway in mendelian randomization, the European most significant contributors include England, which ranks first in terms of the number of articles published, as well as the United States and Canada in North America, China in Asia, and Australia in Oceania.

### 3.2. Analysis of countries

**Figure 3.**
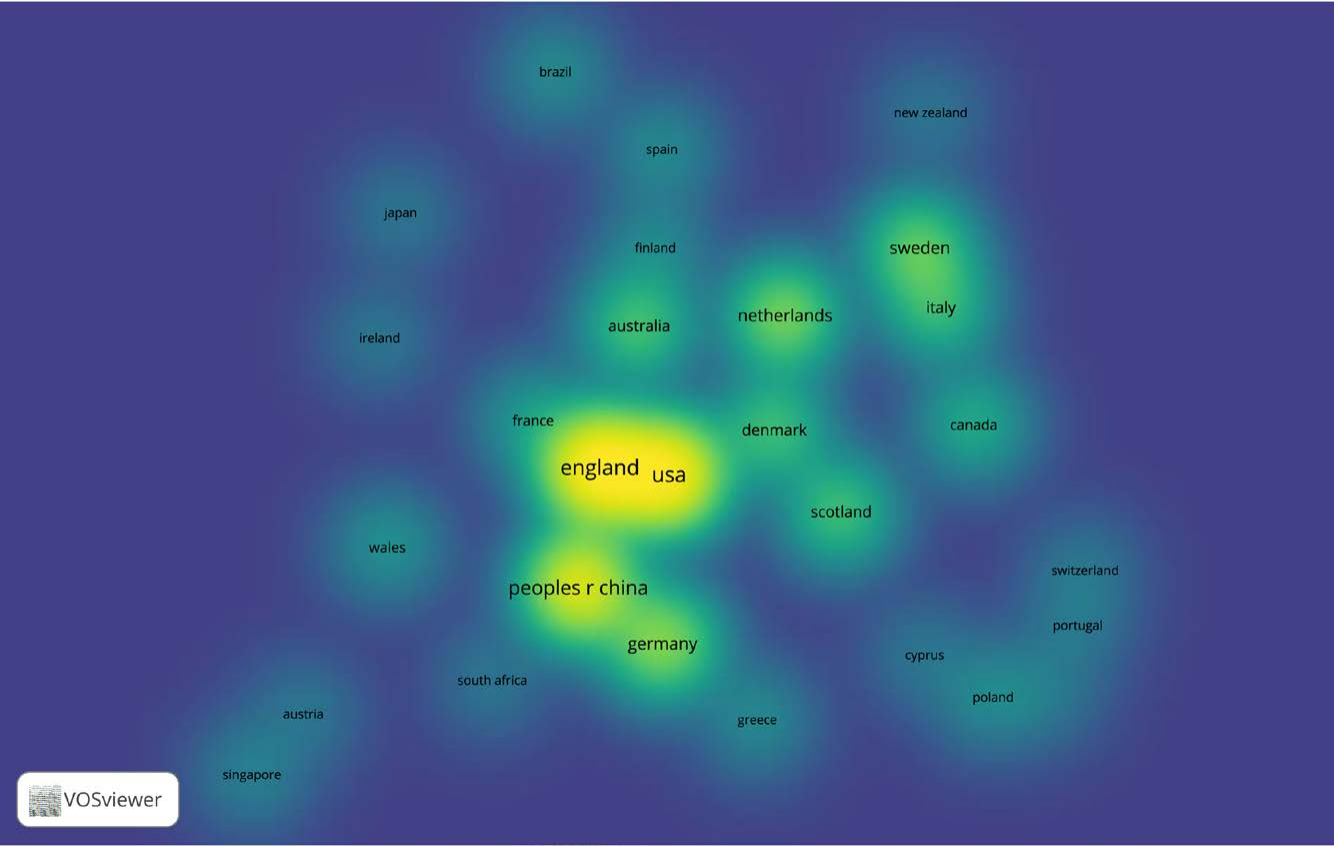
Density visualization of the distribution of all articles across countries (Larger and brighter regions indicate the greatest number of publications in the field of IL-6 signaling pathway in mendelian randomization from a specific country, while closer regions in space represent closer national collaborations) IL-6 = interleukin 6.

The included publications on IL-6 signaling pathway in Mendelian randomization were published in 41 countries. The top 5 countries that contributed to research articles in this field were summarized in Table 2. 39.0% (64/164) articles were published by England, followed by the United States (35.9%; 59/164), China (27.4%; 45/164), Germany (15.2%; 25/164), and Netherlands (13.4%; 22/164) (Table 2), which can also be noted in the density visualization illustrated by Figure 3. It is noteworthy that despite developing a relatively small amount of publications (22) in the top 5 countries, Netherlands embraces the highest average of citations (67.4), revealing its significance in this field. Additionally, Figure 3 also indicates that in fact there is more cooperation between the top 2 countries England and the United States in the field of IL-6 signaling pathway in Mendelian randomization.

**Table 2:** The top 5 countries contributed to research publications in the field of IL-6 signaling pathway in mendelian randomization.

Rank	Country	Publications	Percentage	Average citation
1	England	64	39.0	46.8
2	United States	59	35.9	57.9
3	China	45	27.4	15.6
4	Germany	25	15.2	45.8
5	Netherlands	22	13.4	67.4

IL-6 = interleukin 6.

### 3.3. Analysis of institutions

**Figure 4.**
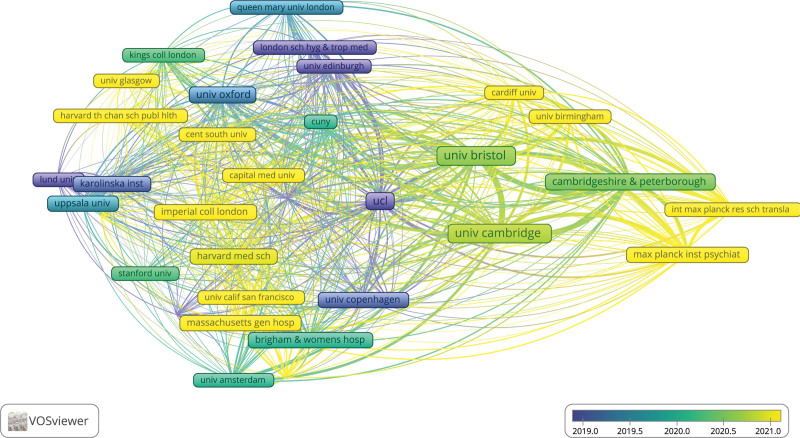
Overly visualization of institutions with different publication periods (The larger the bubbles are, the greatest number of publications of the institution appearing. In terms of time, the brighter the bubble is, the closer the publications issued by specific institutions are to the present time).

406 institutions delivered 164 publications on the IL-6 signaling pathway in Mendelian randomization. As shown in Table 3, the most productive institutions are University of Bristol (25, 15.2%), followed by University of Cambridge (22, 13.4%), University College London (16, 9.6%), Cambridgeshire and Peterborough NHS Foundation Trust (11, 6.7%), and University of Oxford (11, 6.7%). All of the above organizations are from England. According to the overlay visualization of institutions, the majority of institutions issue documents published after 2020 (Fig. 4). Furthermore, the average citation of an organization could contribute to evaluating its influence in the relevant field. Literature, published by University of Oxford, had the highest average citation of 71.5, followed by that of University College London at 50.9, Cambridgeshire and Peterborough NHS Foundation Trust at 43.5, University of Bristol at 42.8, and University of Cambridge at 33.6.

**Table 3:** The top 5 most productive institutions in the field of IL-6 signaling pathway in mendelian randomization.

Rank	Institutions	Country	Publications	Percentage	Average citation
1	University of Bristol	England	25	15.2	42.8
2	University of Cambridge	England	22	13.4	33.6
3	University College London	England	16	9.6	50.9
4	Cambridgeshire and Peterborough NHS Foundation Trust	England	11	6.7	43.5
5	University of Oxford	England	11	6.7	71.5

IL-6 = interleukin 6.

### 3.4. Analysis of keywords

**Figure 5.**
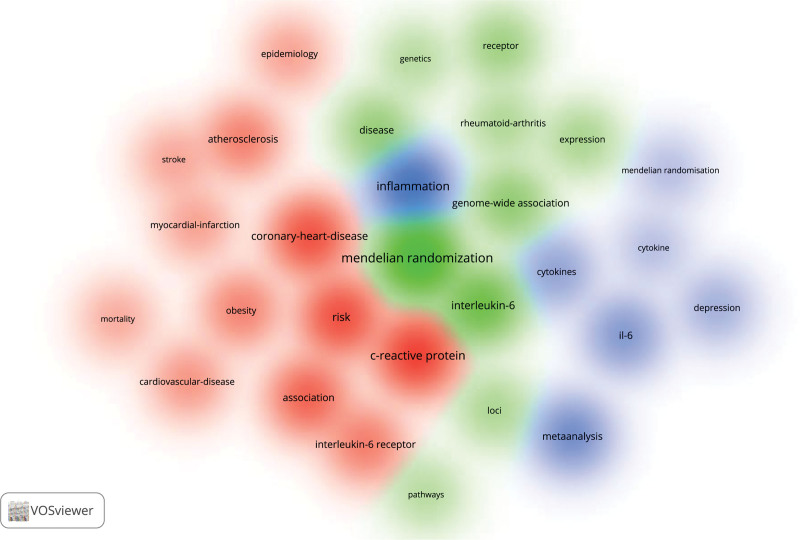
Visualization of co-occurrence keywords (The larger the bubbles are, the higher the probability of the keyword appearing. The different colors of the bubbles represent different clusters, implying that the keyword represented by the same color of the bubble has a stronger correlation).

By investigating co-occurrence keywords, researchers may develop a better understanding of current study hotspots and future development trends. As illustrated in Figure 5, high-frequency keywords in the field of IL-6 signaling pathway in mendelian randomization included mendelian randomization, interleukin-6, il-6, c-reactive protein, association, coronary-heart-disease, inflammation, instruments, risk, and so on. Additionally, the clustering analysis of keywords reveals that the aforementioned high-frequency keywords can be divided into 3 clusters. Cluster 1 (red nodes, 12 items) focuses on IL-6 signaling pathway in cardiovascular diseases, and its keywords include interleukin-6 receptor, association, coronary-heart-disease, myocardial-infarction, risk, c-reactive protein, and so on; Cluster 2 (green nodes, 10 items) focuses on genomic analysis involving IL-6 signaling pathway in mendelian randomization, and its keywords include mendelian randomization, interleukin-6, genetics, genome-wide association, pathways, expression, and so on; Cluster 3 (blue nodes, 7 items) focuses on cytokines of IL-6 signaling pathway in mendelian randomization, with the keywords mendelian randomization, il-6, inflammation, cytokines, and so on (Fig. 5).

### 3.5. Analysis of journals

**Figure 6.**
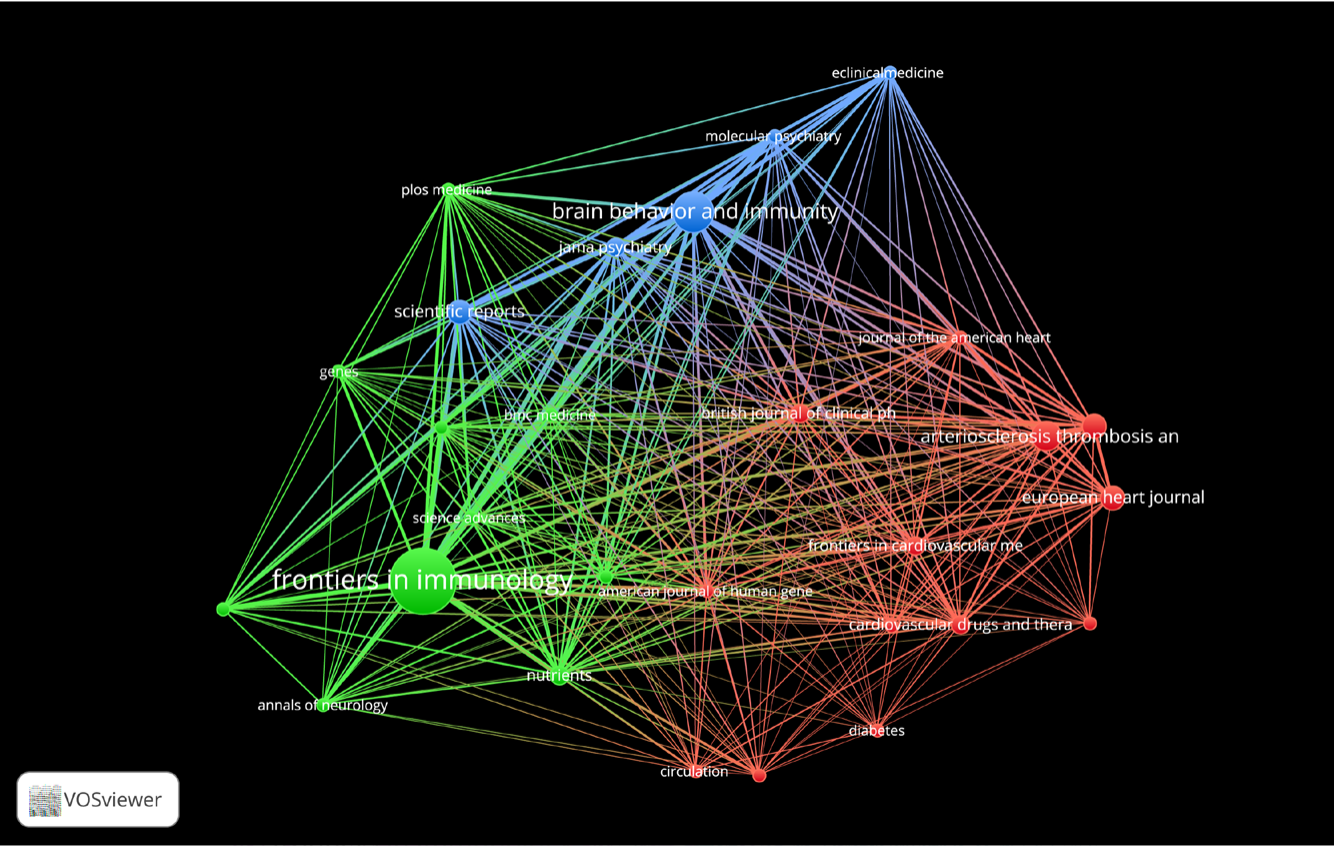
Visualization of published journals (A larger square frame indicates that the greatest number of publications were published by this journal).

These articles included in the field of IL-6 signaling pathway in mendelian randomization were published in 107 journals. The network visualization of journals is displayed in Figure 6 where the quantity of articles published is represented by the size of the node. Moreover, the top 5 productive journals are displayed in Table 4. Frontiers in Immunology has published the most literature (12 articles, 7.3%), followed by Brain Behavior and Immunity (7 articles, 4.3%), Arteriosclerosis Thrombosis and Vascular Biology (5 articles, 3.0%), Circulation Research (4 articles, 2.4%), and European Heart Journal (4 articles, 2.4%). It is worth noting that all the top 5 productive journals were divided into the Q1 (JCR Partition). When it comes to average citations, literature, published by Circulation Research, had the highest average citation of 194.25, while the lowest, 2.5, was reported in literature published by Frontiers in Immunology, which published the majority of the articles.

**Table 4:** The top 5 productive journals in the field of IL-6 signaling pathway in mendelian randomization.

Rank	Journal	Documents	Percentage	Average citation	IF(2023)	JCR(2023)
1	Frontiers in Immunology	12	7.3	2.5	7.3	Q1
2	Brain Behavior and Immunity	7	4.3	64.4	15.1	Q1
3	Arteriosclerosis Thrombosis and Vascular Biology	5	3.0	10.6	8.7	Q1
4	Circulation Research	4	2.4	194.25	20.1	Q1
5	European Heart Journal	4	2.4	162.0	39.3	Q1

IL-6 = interleukin 6.

### 3.6. Analysis of authors

**Figure 7.**
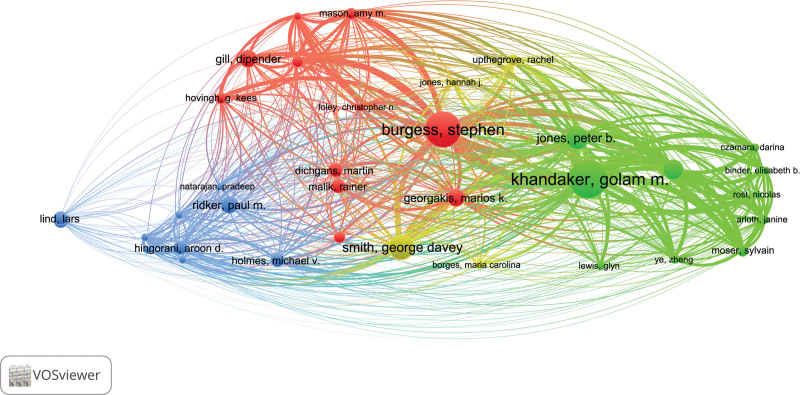
Cooperation network of productive authors (The map bubbles represent authors, and the lines connecting the bubbles represent collaborative relationships. The larger the bubble area, the greater the number of publications).

981 authors contributed to the selected 164 publications. The top 5 high-yield authors are listed in Table 5. The most productive author is Golam M Khandaker (n = 13), followed by Stephen Burgess (n = 12), George Davey Smith (n = 8), Peter B Jones (n = 7), and Nils Kappelmann (n = 6). Additionally, combined with the Total Link Strength illustrated in Table 5, Figure 7 shows that Golam M Khandaker, Stephen Burgess, Peter B Jones, and Nils Kappelmann have closer cooperation with each other, which can also be observed in an article titled “Associations of immunological proteins/traits with schizophrenia, major depression and bipolar disorder: A bi-directional 2-sample mendelian randomization study.”

**Table 5:** The 5 high-yield authors in the field of IL-6 signaling pathway in mendelian randomization.

Rank	Author	Publications	Average citation	Total link strength	Country
1	Golam M. Khandaker	13	38.9	5046	England
2	Stephen Burgess	12	38.8	4773	England
3	George Davey. Smith	8	63.6	2521	England
4	Peter B. Jones	7	48.3	3331	England
5	Nils Kappelmann	6	42.0	3169	Germany

IL-6 = interleukin 6.

### 3.7. Analysis of co-cited references

**Figure 8.**
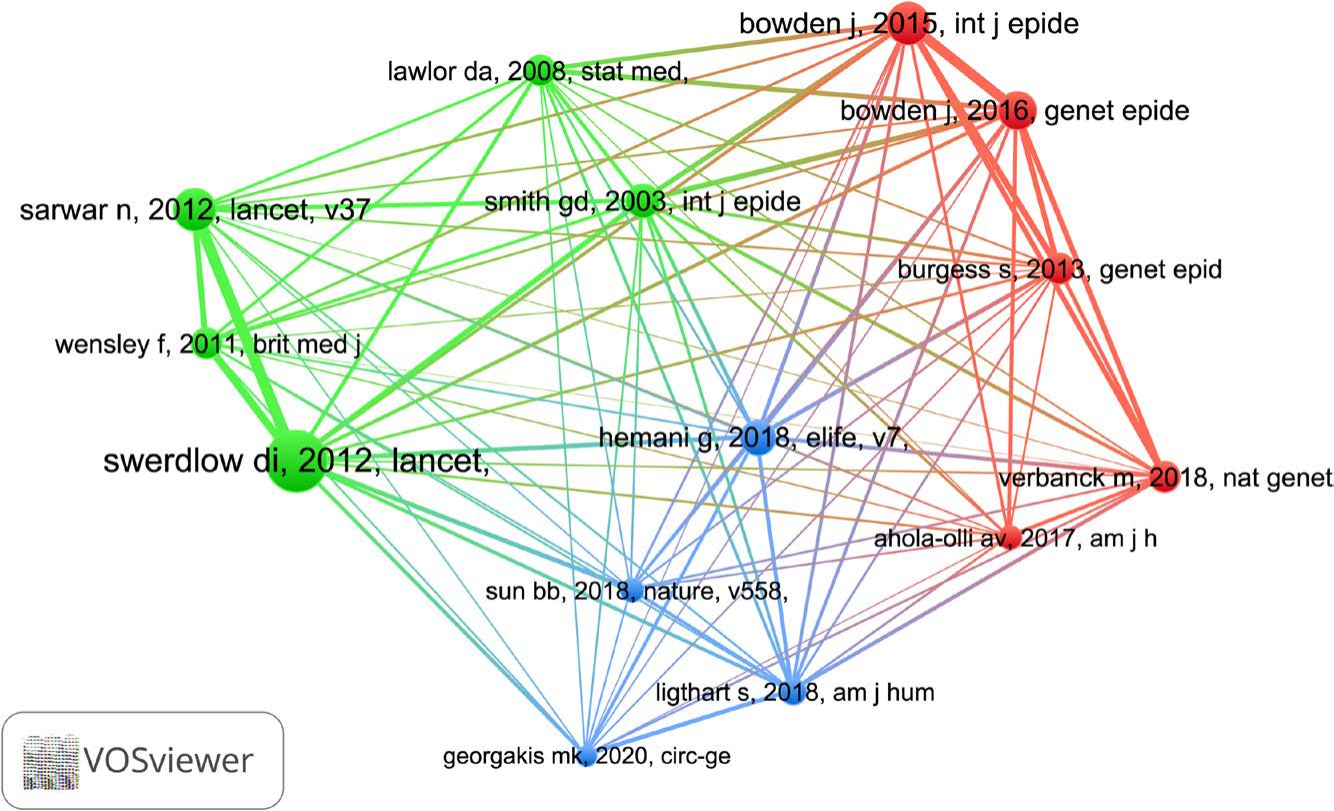
Visualization of co-cited references (The larger the bubble, the more the literature is likely to be co-cited. The different colors of the bubbles represent different clusters. Differences in the topics covered by these co-cited references are indicated by different clusters).

The network visualization of co-cited references, generated with VOSviewer, is shown in Figure 8. Among 7385 cited references, the minimum number of citations set to at least 20, and finally, 14 references were included in this analysis. Furthermore, the network visualization of co-cited references co-occurrence analysis revealed that the references are mainly divided into 3 clusters. In the green cluster, mendelian randomization was employed to examine the role of the IL-6 signaling pathway in cardiovascular disease. The literature on red cluster was mainly concerned with the theoretical instrumental study of Mendelian randomization. Whereas the literature on blue cluster focused on genome-wide analyses in Mendelian randomization. In addition, Table 6 listed the top 5 co-cited references in the field of IL-6 signaling pathway in Mendelian randomization.

**Table 6:** The top 5 co-cited references in the field of IL-6 signaling pathway in mendelian randomization.

Rank	Author	Title	Yr	Journal	Citations
1	Daniel I Swerdlow	The interleukin-6 receptor as a target for prevention of coronary-heart-disease: a mendelian randomization analysis	2012	lancet	66
2	Jack Bowden	Mendelian randomization with invalid instruments: effect estimation and bias detection through Egger regression	2015	International Journal of Epidemiology	45
3	Nadeem Sarwar	Interleukin-6 receptor pathways in coronary-heart-disease: a collaborative meta-analysis of 82 studies	2012	Lancet	45
4	Jack Bowden	Consistent estimation in mendelian randomization with some invalid instruments using a weighted median estimator	2016	Genet Epidemiol	41
5	Gibran Hemani	The MR-base platform supports systematic causal inference across the human phenome	2018	Elife	38

IL-6 = interleukin 6.

## 4. Discussion

The present study focuses on the results of a bibliometric analysis of 164 publications on the IL-6 signaling pathway in mendelian randomization between January 2013 and August 2023 utilizing the WoSCC database, VOSviewer software, Microsoft Excel 2021, and Scimago Graphica. To the best of our knowledge, this is the first bibliometric analysis of research on Mendelian randomization of IL-6 signaling pathway in related diseases. Combined with publication trends, the discipline of IL-6 signaling pathway in mendelian randomization has significantly advanced since 2020. Over the next few years, it is expected that there will be a constant increase in publications in this field, which might provide researchers working to examine the role of the IL-6 pathway in associated disorders more hotter research ideas.

The country with the most publications is England, which is followed by the United States and China, both of which have also made significant contributions to this field. According to the visualization of VOSviewer software, the collaboration between England and the United States is more frequent. For instance, research by Georgakis, Marios K. (the United States) and Burgess, S. (England) reveals that IL-6 signaling that is genetically downregulated is linked to a lower lifetime risk of cardiovascular disease.^[21]^ Also, Dantzer, Robert from the United States and Khandaker, Golam M. from England both published an article titled Interleukin-6 as potential mediator of long-term neuropsychiatric symptoms of COVID-19.^[22]^ In addition, it is noteworthy that top 5 most productive institutions are all from England, which indicates that England holds a significant position in this field when combined with previous information revealing that England published the majority of documents.

Academic journals serve as both the main source for cutting-edge knowledge in a specific discipline and a platform for researchers to publish their relevant results. Based on the co-occurrence analysis of the published journals, we identified a total of 106 journals that have published articles in this field of IL-6 signaling pathway in mendelian randomization. The scientific journal with the majority of publications is Frontiers in Immunology, which covers research on the molecular mechanisms of various immune-related illnesses. It should be noted that 2 (Frontiers in Immunology and Brain Behavior and Immunity) of the top 5 productive journals address research on immune diseases and the remaining 3 (Arteriosclerosis Thrombosis and Vascular Biology, Circulation Research, and European Heart Journal) deal with cardiovascular disease is seemingly of significance given that the IL-6 pathway in the mendelian randomization is primarily involved in cardiovascular^[23,24]^ and immune-related diseases.^[25,26]^ In terms of authors, Golam M Khandaker is the author with the highest number of publications and, surprisingly, most of her articles are dedicated to the field of psychiatry, investigating the relationship between the IL-6 signaling pathway and psychiatric disorders such as depression and anxiety.^[27–29]^ The author with the second-highest number of publications, Stephen Burgess, whose research focuses mostly on the interaction between the IL-6 signaling pathway and cardiovascular disease.^[24,30]^

Keyword co-occurrence analysis can help researchers better identify current research hotspots and future research trends. As shown in Figure 5, The most common keywords in the field of IL-6 signaling pathway in mendelian randomization are “mendelian randomization,” “interleukin-6,” “il-6,” “c-reactive protein,” “association,” “coronary-heart-disease,” “inflammation,” “instruments,” “risk,” “rheumatoid arthritis,” “depression.” Cluster analysis of the keywords also revealed that mendelian randomization involving IL-6 signaling pathway was mostly related to cardiovascular diseases. According to the research by Yuan et al, IL-6 signaling pathway inhibition may offer novel approaches to preventing disorders like coronary artery disease, atrial fibrillation, and ischemic stroke.^[24]^ Of course, the IL-6 signaling pathway has also been associated with other diseases, including depression,^[16]^ multiple sclerosis,^[25]^ low back pain,^[31]^ systemic lupus erythematosus,^[18]^ ischemic stroke,^[32]^ rheumatoid arthritis,^[17]^ asthma,^[33]^ etc. Taken together, current research hotspots in this field focuses on the application of Mendelian randomization to investigate the forms of association between IL-6 signaling pathway and cardiovascular, neurological, immunological and inflammatory diseases. Moreover, the future trends in this field is to further explore the association of the IL-6 signaling pathway in new diseases associated with the above using Mendelian randomization.

## 5. Limitations

There are several limitations to the research. As with prior bibliometric analysis, firstly, some of the pertinent literature had unavoidably been left out because the data in this study solely was derived from WoSCC database. Secondly, only documents in English were included. it possible that contributions in other languages went unnoticed. Despite the limitations mentioned above, we believe the outcomes of this scientific study can still reflect the general research status and development trend of the field based on the authority of the WoSCC database.

## 6. Conclusion

In this study, the knowledge structure and research frontiers in the field of IL-6 signaling pathway in mendelian randomization are thoroughly summarized employing bibliometrics and visualization analyses, which also identify current research hotspots and future perspectives. The number of publications in this area has increased significantly since 2020, indicating that research related to this area has attracted significant interest in recent years. England and the United States were the most active participating countries, while the Frontiers in Immunology was the most active sources of publication for this research filed. Keyword analysis results identified “mendelian randomization,” “inflammation,” “interleukin-6,” “c-reactive protein,” “coronary-heart-disease” as the top 5 most frequently used keywords that reflect current hotspots and prospective future trends in this field of research.

## Author contributions

**Conceptualization:** Shaoze Jing.

**Data curation:** Shaoze Jing.

**Formal analysis:** Shaoze Jing, Jiani Wang.

**Investigation:** Jiani Wang.

**Methodology:** Jiani Wang.

**Resources:** Shuhan Yang.

**Software:** Shuhan Yang.

**Supervision:** Hua Wu.

**Writing – original draft:** Shaoze Jing, Shuhan Yang.

**Writing – review & editing:** Hua Wu.
